# Prototyping a precision oncology 3.0 rapid learning platform

**DOI:** 10.1186/s12859-018-2374-0

**Published:** 2018-09-26

**Authors:** Connor Sweetnam, Simone Mocellin, Michael Krauthammer, Nathaniel Knopf, Robert Baertsch, Jeff Shrager

**Affiliations:** 1 Cancer Commons, Los Altos, CA USA; 2 Istituto Oncologico Veneto, IOV-IRCSS; and Department of Surgery Oncology and Gastroenterology, University of Padova, Padova, Italy; 3 Program for Computational Biology and Bioinformatics, Yale University, New Haven, CT USA; 4 Department of Pathology, Yale University School of Medicine, New Haven, CT USA; 5 EECS Department, MIT, Cambridge, MA USA; 6 Symbolic Systems Program, Stanford University (Adjunct), Stanford, CA USA

**Keywords:** Natural language processing, Precision oncology, Controlled natural language, Nanopublication, Treatment reasoning, Rapid learning, Tumor boards, Targeted therapies

## Abstract

**Background:**

We describe a prototype implementation of a platform that could underlie a Precision Oncology Rapid Learning system.

**Results:**

We describe the prototype platform, and examine some important issues and details. In the Appendix we provide a complete walk-through of the prototype platform.

**Conclusions:**

The design choices made in this implementation rest upon ten constitutive hypotheses, which, taken together, define a particular view of how a rapid learning medical platform might be defined, organized, and implemented.

## Background

Shrager and Tenenbaum [25] described a closed loop system where case-based treatment and outcome information from across the cancer community flow into a knowledge base that is then directly used in decision support. Treatment choices and outcomes based on this decision support, flow back into the knowledge base, creating a process that Shrager and Tenenbaum termed “Rapid Learning Precision Oncology”, or “Precision Oncology 3.0” (PO 3.0). The present paper describes a prototype implementation of a platform that could support a PO 3.0 rapid learning system.

The decision support application chosen for our prototype is based on Mocellin et al.’s [16] “Targeted Therapy Database” (TTD). They asked a handful of melanoma physicians and researchers to manually summarize peer-reviewed articles describing anti-melanoma targeted therapies. These experts extracted what we will hereafter call the “Treatment Rationales” (TRs), either explicitly or implicitly expressed in each paper. Mocellin et al. also proposed an algorithm to rank therapeutic hypotheses based upon the molecular profile of a putative patient’s tumor. Importantly, this algorithm weighed each piece of information in the 2010 TTD by the level of evidence: preclinical (cell line, animals) and clinical (case report, clinical trials phase I - II - III, meta-analysis of randomized trials and so on). The theory of the 2010 TTD is that combining a wide variety of clinical and preclinical information regarding any type of molecular feature (alterations, mutations, expression, perturbation, variations, and so on), and drug sensitivity or resistance, can be very informative.

Unfortunately, keeping the 2010 TTD up to date would require continuous attention from melanoma domain experts, and if the database was expanded beyond melanoma, keeping it current would quickly become intractable. Moreover, because it concentrated on published knowledge, the 2010 TTD did not incorporate information about the clinical improvisations that clinicians undertake in their daily efforts to treat the many advanced cancer patients who have progressed beyond the standards of care, but cannot find an appropriate trial, as this information is mostly unpublished.

Stevovic et al. [26] proposed a novel approach to this problem. They suggested augmenting the 2010 TTD with information derived from patient cases, which is a narrow, although clear and (theoretically) easily accessible source of information about these state-of-the-art clinical improvisations. Under the Stevovic et al. hypothesis, by virtue of adding case experience to the 2010 TTD, treatment rankings produced by the Mocellin et al. algorithm, would automatically combine findings from the literature with findings from experiments carried out on individual cases. Thus, continuous capture of rich case-based experience into the TTD would, Stevovic et al. reasoned, keep the knowledge base up to date in the desired manner.

In the present paper we describe a prototype implementation of a platform that could underlie a PO 3.0 Rapid Learning system, as envisioned by Shrager and Tenenbaum [25]. In creating this prototype we have extend the concepts discussed above, particularly numerous enhancements to capture case-based information, and made numerous concrete decisions where the previously cited papers left many details unspecified. The choices we have made are based upon ten constitutive hypotheses. In the next section we explain these hypotheses, and then, in the following section, describe the prototype implementation. Then we take up some important issues and details. A complete walk-through of the prototype platform is described in Appendix, and it can be tested online.

### Constitutive hypotheses

The choices we have made in the design of our prototype PO 3.0 platform arise from a set of ten constitutive hypotheses. These hypotheses are closely interrelated, but to point out every such relationship would make the presentation too complex. Therefore, although we have tried to point out some of these relationships, many are left to the reader’s own reason.

#### The central motivating hypothesis

##### H1. The Case Efficiency Hypothesis

This is our central motivating hypothesis. In almost all circumstances, more information is better than less. So it almost goes without saying that collecting information about naturally-occurring, un-coordinated, off-trial cases would be more useful than not collecting this data. Of course, anyone reasoning from this data must take into account that it is not prospective, randomized, nor controlled. In describing PO 3.0, Shrager and Tenenbaum [25] assert that a large number of *prospective, adaptively coordinated* individual case studies can be as statistically strong, or perhaps even stronger, and more efficient than large scale, randomized, controlled clinical trials. If this is the case, then (by hypothesis) one does not need to run large scale trials at all, but rather just efficiently coordinate and collect all (or at least many) such experiments, carried out across the clinical oncology community. (In the “real world”, of course, there is now, and will likely always be, a combination of large scale trials, as well as retrospective and prospective database studies, although most off-trial case data is locked up in EHRs, and anyway would be from uncoordinated treatment experiments.) In the present paper we have not set out to test this hypothesis, but rather to design and prototype an informatics platform that could support PO 3.0, and thereby support a test of the Case Efficiency Hypothesis.

#### Hypotheses about knowledge and where to find it

##### H2. The Treatment Rationale Hypothesis

We shall hereafter use the term “Treatment Rationale” (TR) to mean the reason (i.e., an explanation) that a particular course of action (e.g., test, treatment, watchful waiting, etc.) was either recommended or rejected (i.e, contra-indicated). A useful, although very narrow, subset of TRs are the rows in [16] TTD, and, as will be seen, these are the core information gathered by our PO 3.0 platform prototype. Therefore, for present purposes, we use the term “TR” as a shorthand for TTD entries. Later we will discuss the broader class of general TRs.

The TRs collected by Mocellin et al. were extracted by domain experts from published papers. Stevovic et al. proposed augmenting the 2010 TTD with TRs that are reported by patients themselves. We are focused on yet a third method, extracting TRs from experts in the context of live, case specific reasoning. Regardless of where they come from, all the TRs that we will deal with have the same form. In the 2010 TTD, the TRs represented the treatment hypothesis that was expressed, or in some cases implicit, in the summarized paper. Stevovic et al. would have the TRs arise from a description of the patient molecular data, the treatment tried, and the observed outcome. We propose a more subtle version of a Treatment Rationale that emphasizes the *explanation* of the actual (or supposed) reason that particular treatments or classes of treatments were recommended or excluded (i.e., indicated or contraindicated). Treatment Rationales as *explanations for treatment recommendations* represent the core content of clinical reasoning, providing the inferential connection between observations and decisions. TRs are the products of clinical assessment by domain experts (e.g., clinicians or scientists or groups of these), and codify the justification for a selection of treatment options applicable to the patient being treated, based upon some presumably relevant characterization of the state of the patient’s disease. In addition to molecular characteristics of a tumor, TRs will often include other factors such as economics (e.g., the costs of the treatment, the patient’s insurance), patient preference, ethical factors, and even a physician’s personal experience or taste. These factors are very hard to capture if one is looking merely at measured outcomes, and very difficult to organize clinical trials around.

It is critical to recognize the importance of capturing rationales not only for the recommended treatment (test, etc.), but especially for those that are considered but set aside, or given lower ranking, because they are either incorrect, undesirable, or infeasible. These “contra recommendations” carry as much, or in some cases more, information than the final recommendation that is carried out of a tumor board; Often the actual recommendation is a “safe” or “possible” choice, whereas a physician might *like* to do is something that *may* be more effective if it weren’t for practical barriers, such as cost, side effects, or patient preference. Even more importantly, such contra recommendations may represent new treatment hypotheses, possibly worthy of testing in a trial. Our hypothesis is that capturing both pro- and contra-recommendations will enable us to create a richer understanding of the determinants of clinical cancer care, and thereby provide richer and more accurate decision support. (In the platform prototype, described later, we will see that the Treatment Explorer tool, TrEx, utilizes both positive and negative evidence, analogous to pro- and contra-recommendations.)

As mentioned at the beginning of this section, TRs, as conceived here, do not capture every possibly relevant aspect of clinical reasoning, but our hypothesis is that it is critical to have such explanations in order to correctly and efficiently learn the applicability rules for particular actions in particular contexts, especially in high-dimensionality domains such as molecular biomedicine [15]. We will talk more about the more general category of TRs in the discussion section, below.

##### H3. The Abundance of Outcomes Hypothesis

It is usually assumed that one needs to have final outcomes in order to develop clear treatment choice statistics, and that in the best case one would have well-powered, randomized, controlled trials whose primary data represents long-term survival. We argue that this requirement is too strong, leading to the loss of an abundance of data that includes both direct and indirect outcomes. Direct outcomes can come from long-term survival data (whether in an individual case, or in a trial context), but are often more easily accessible in short term proxy treatment responses, such as a slowing of disease progress measured by, for example, reduction in tumor load. Although these are not complete replacements for overall survival, as with the Case Efficiency Hypothesis, these are data, which if interpreted correctly, and with an understanding of their limitations, should not be ignored. Another easily available source of direct outcomes is simply the patient history, i.e., a patient showing up for second, third, or fourth line treatment has, by definition failed (and perhaps temporarily succeeded) on previous treatments. These are valuable outcomes data.

But there are also *indirect* outcomes data to be found merely in expert treatment choices, and especially in Treatment Rationales, defined above as explanations. Domain experts’ choices, and their explanations for these choices, contain indirect outcome data because experts, by definition, have “good reasons” for making choices, and these “good reasons”, encoded in TRs, bring together retrospective outcome information, whether published, personally experienced, or even hearsay.

This brings us directly to the next hypothesis:

##### H4. The Expert Focus Hypothesis

Rather than summarize the past decade of published oncology TRs, as was done by Mocellin et al., we are here proposing, instead, to capture TRs from experts in the process of reasoning about real patients -- i.e., to capture “case-contextualized TRs”. Below we will take on the claim that reasoning should be captured in context of real case reasoning. Here we focus on why we want to get knowledge from experts, rather than, for example, the peer reviewed literature, any physician, relevant patients, or at the other extreme, even just anyone with an opinion (i.e., crowdsourcing).

As mentioned briefly above, much of what experts know and use in regular decision-making is not published (e.g., it comes from their personal experience and knowledge), and may not even be practically or ethically testable in clinical trials. Many experiments are either impractical, impossible, or unethical to carry out, and as we have found out more and more recently, at least some, and possibly most, of what is published is wrong [8].

Indeed, the use of personal experience is explicitly codified in most analyses of the levels of evidence appropriate for medical decision making, for example:

“First, physicians should offer standard care. If no standard care option exists, the physician should consider enrollment into a randomized clinical trial. If no trial is appropriate, the physician should consider a non-randomized registry, or consensus based guidelines. Finally, only after considering the first three options, the physician should use best judgment based on previous personal experience and any published case series or anecdotes. Given the paucity of quality randomized clinical trial data for most medical decisions, the ‘best judgment’ option will be used most frequently” [14].

The Expert Focus Hypothesis does not imply that experts are the *only* sources of information that one requires, nor does it include the assertion that experts are always correct. The core claim is just that expert knowledge (as represented here by case-contextualized TRs) is an efficient way to focus the Precision Oncology 3.0 process.

##### H5. The Case Context Hypothesis

The Expert Focus Hypothesis (H4) asserts that TRs should be captured from experts. The Case Context Hypothesis asserts that they should be captured in the context of real case reasoning. There are several important reasons for this claim. First, the case context provides the provenance for the TRs, analogous to a literature citation. Second, the case context, and, to the extent possible, surrounding discussion, are often important in understanding the meaning and limitations of TRs, and for assessing their validity and generality. Many scholars believe that knowledge deployed in an active reasoning context (i.e., “dynamic” knowledge) differs in important ways from knowledge found in papers and books (i.e., “static” knowledge) (e.g.,[9, 10]). Although it is not entirely understood why this difference exists, because of it, the context in which reasoning takes place is often critical to correctly interpreting and using what was said.

##### H6. The Problem-Solving Tumor Board Hypothesis

Taken together, H3, H4, and H5 strongly suggest that TRs cannot come from patients, as was proposed by Stevovic et al., but must come from clinicians or other domain experts, as was the case for Mocellin et al. This suggests that case-contextualized TRs must come from *published* case reports. (And indeed, there were some examples of such case reports in the knowledge base for the 2010 TTD.) However, this requirement would be too limiting in light of The Case Efficiency Hypothesis (H1) which assumes that all, or at least a substantial fraction, of case knowledge is captured. Only a miniscule fraction of cases that could represent useful Treatment Rationales actually end up in published case reports, as it requires significant effort to write up a case, beyond all the effort involved in the actual practice of medicine, and current incentive structures do not significantly reward this effort.

Although treatment rationales regularly arise in live clinical problem solving, and are regularly voiced in discussion among physicians, such rationales rarely end up in medical records, and are almost never shared in detail with patients. This is particularly true for *rejected* treatment hypotheses and the reasons for their rejection, even though, as argued above, these may be critical for understanding the pros and cons, and eventually the success or failure, of various treatment options (e.g., [30]). For all of these reasons, we must look beyond formal publications, and beyond patient-supplied information, for case-contextualized TRs.

Fortunately, the reasoning underlying clinical problem-solving about difficult cases is commonly discussed in the expert problem-solving settings that we will refer to as “problem-solving Tumor Boards”^1^ (psTBs), which are now commonly mounted at major cancer centers [End note 1]. In creating treatment recommendations, psTBs integrate a wide range of knowledge, including that mentioned above as forming the content of TRs, such as: details of tumor omics, histopathology, clinical history, patient preferences, economics, ethics, and other factors. psTBs often consult experts in bioinformatics, genomics, molecular biology, and other domains. As a result, the content of psTB discussions often contains unique knowledge that may significantly inform cancer research and clinical care. Moreover, as previously mentioned, knowledge dynamically deployed in the live problem-solving contexts, such as in psTBs, is often not the same as the static explicit knowledge available directly from papers.

Unfortunately, the knowledge deployed during psTB reasoning is rarely captured and communicated in either case reports or case series. More often than not, the relevant information, if it is recorded at all, ends up in an EMR (and often just in the notes), where it may eventually serve as evidence in an aggregated analysis. But EMR records rarely include the richness of clinical reasoning typical of the psTB discussion, or of published case reports, and in particular are unlikely to include the reasons that some treatments were considered but reasoned to be *contraindicated*, and so rejected.

We hypothesize that clinical reasoning captured from live psTB discussions is a valuable new source of knowledge. Among other functions, the capture of TRs from psTBs may help to focus the hypotheses tested in clinical trials, reducing the number of trials required to efficiently search treatment space. psTB reasoning may also help to guide the development of tools to assist in clinical decision-making. And at the very least can serve as expert reasoning models for clinical education. Moreover, psTBs see only the most difficult cases -- usually cases where the patient has exhausted the standard of care, and often cases for which there is no appropriate clinical trial.

For all of these reasons, we have focused our prototype design on capturing, vetting, and sharing de-identified case summaries and case-contextualized TRs, sourced from psTBs.

#### Hypotheses arising from the air traffic control analogy

Shrager and Tenenbaum [25], also Shrager [22] proposed Global Cumulative Treatment Analysis (GCTA) as a method for efficiently searching the huge space of treatments crossed with patient tumor characteristics. Recently, Shrager [23, 24] described GCTA through the analogy of an adaptive “Air Traffic Control”-like coordination of treatment choices across all patients in the cancer community. This analogy provides several useful hypotheses.

##### H7. The Coordination Over Collaboration Hypothesis

Calls for better collaboration in the biomedical community are commonplace. However, many complex systems organize themselves efficiently by *coordination* rather than collaboration. Coordination, in this context, amounts to indirect collaboration. In commercial aviation, for example, pilots are strongly discouraged from talking to other pilots; That just fills the airwaves with chatter. Instead pilots talk almost exclusively to controllers. The controllers have a larger view of what’s going on in the system, and can coordinate traffic by guiding the pilots with recommended actions. It is, of course, the pilots who have their hands on the actual controls, and make the final decisions. But collaboration between pilots usually takes place indirectly as a result of the pilot-controller interaction.

It is important not to confuse the sort of “between-team” collaboration we are discussing here, with what might be called “within-team” collaboration, for example among the pilots and crew on a single airplane, or among physician and nurses on a single care team, or even among multiple professionals working the same case. Of course, the participants in all of these “within-team” cases collaborate closely, as do controllers on the ground with one another, and the pilots can be said to be collaborating with the controllers. On the other hand, what does *not* regularly happen in the Air Traffic System is pilots directly collaborating with pilots *on other planes*; Or at least this happens only very rarely. Our hypothesis is that this sort of “between-team” collaboration should take place instead via coordination, mediated by a third-party that has a larger view of the system, not by physicians talking to one another across sensible team boundaries.

There is a philosophical question raised by this hypothesis about how to define a “team”. For example, one might say that “all of medicine is on the same team, fighting against disease”, and thus there should then be free flow of cross-communication. Once again, we think of the Air Traffic Control analogy. Of course, in a sense, all the pilots and controllers all over the world are on the same huge “team” in the vague sense of making air travel safe, etc. However, if all the pilots were talking to one another directly, as opposed to interacting via coordinating controllers, the air waves, not to mention the air space, would be chaos! For us the question of where there should be coordination comes down to the very practical question of when a coordinating entity (e.g., controllers) are useful in avoiding multi-way communication chaos. Or, put more positively, a coordinating entity can often improve the efficiency of a complex system by guiding participant actions across the system based upon their larger available view of system activity. To bring this back to cancer, Shrager and Tenenbaum [25], in their GCTA proposal (and [22–24]), hypothesized that efficiently searching the huge space of treatments crossed with patient tumor characteristics, will require a coordination model akin to the way that Air Traffic Control coordinates all commercial aviation. What precisely this authority should be, and how precisely it should operate is work for the future. What this hypothesis implies for our prototype is that it should run as a cloud-based platform where all of the participants are putting in, and using, the TRs, and other content. One might well, ask: “Okay, so cloud-based almost goes without saying these days ... Versus what?” Our answer is, specifically: “Versus physicians collaborating directly with one another (e.g., across tumor board lines).” We think that that would be a recipe for chaos in the larger scale, and would reduce, rather than increase, the efficiency of treatment validation. Therefore, we are specifically *not* building a communications infrastructure, such as a new form of email, texting application, or forum.

##### H8. The Biomedical Controlled English Hypothesis

Most readers, and most airline passengers, will probably be surprised to learn that all commercial pilots and air traffic controllers everywhere in the world communicate in English [7]. Readers (and passengers) will probably be less surprised to learn that the language that pilots use to communicate with controllers is highly stylized.

“What we say and the way we say it make aviation communications unique. It is precise and when correctly performed is designed for clarity and understanding. [...] The use of excess verbiage greatly reduces clarity. [...] Don’t use unnecessary phrases or politeness. [...] Learn the standard FAA phraseology and use it. [...] Use telegraphic brevity. Give ALL the required information only. [...] Learn when it is appropriate to supply ATC with information. [...]” [20].

ATC communications are designed to be efficient and unambiguous. Biomedical communication has similar goals, so our platform focuses on what we will call “Biomedical Controlled English” (BCE), a biomedically-specific form of Controlled Natural Language (CNL; [11]).

CNL is a domain-specific subset of natural language that is obtained by restricting the grammar and vocabulary in order to reduce or eliminate ambiguity and complexity. CNLs are commonly utilized by industry and the military to write manuals and other texts that can be automatically translated into many different languages [2]. One of the important advantages of CNL is that it is easily readable by humans, and also enables correct “compilation”, that is, automatic translation to a formal representation for computational analysis. Because CNL is a version of a natural language, humans can read and check it. Moreover CNL can capture essentially anything that can be said in a natural language. In software engineering terms, CNL a “lossless formal language”. In writing CNL, you do not have to drop any information, as would almost certainly be done in any sort of translation into a non-CNL formal language. As a result of these properties, physicians can vet TRs expressed in BCE (i.e., our biomedical CNL), and at the same time the data can be automatically compiled, without loss of the source-level information, into special-purpose internal formats for any of a wide range of computational analyses. These special-purpose internal formats can be retain much information as necessary because the BCE is always retained, and it contains all the original information.

#### Hypotheses about knowledge publication

##### H9. The Nano-Publication Hypothesis

Rapid Learning demands a fast and flexible communication channel. In our discussion above of the Coordination Over Collaboration Hypothesis (H6), we pointed out the need to have a rapidly updated, common (therefore cloud-based) knowledge base of vetted TRs, being fed from every psTB via case-contextualized BCE TR capture, and feeding into every psTB via decision support tools. (More on vetting later.) One way to do this is to simply put the vetted TRs online on an open web site, but this has issues with archive stability and provenance. A better developed method is offered by “nano-publication” [13, 28], wherein linked data, API access, versioning, automatic re-distribution, provenance, and other desirable properties can be provided. Even though we believe that nano-publication is the correct choice for publishing TRs, we have chosen, in the present prototype, to create our own local pseudo-journal in the style of nano-publication. The reason that we have taken this halfway step is merely to protect the “live” nano-publication infrastructure, an existing set of linked servers providing access to millions of nano-publications [13], from what will, for some time, be dummy data deriving from our prototype. In a live deployment we expect to push vetted TRs out to the real nano-publication system.

##### H10. The BCE Publication Hypothesis

In what format should the TRs be published in the nano-journal? If one takes the CNL hypothesis seriously, and believes that BCE is the sweet-spot between natural conversation and some deep semantic representation, then the publications should appear in BCE, and then can include any number of additional annotations and translations. There is a good practical reason to focus on the BCE form of TRs: These are the format that has been vetted. Moreover, these are, as described above, the only guaranteed lossless format, whereas any (current) version of NLP compilation that tries to create a formal representation from the BCE will necessarily lose information. Therefore, we hypothesize that one always wants to store and publish the original, vetted, lossless BCE. ([12] make a similar argument.)

In the present work, we translate (compile) the TRs from BCE into TTD-like assertions. But this is specific to our Treatment Explorer application (TrEx, as described below); There are an infinitude of possible translation (compilation) target applications. For example, an analysis that tags concepts with ontological identifiers may facilitate deep semantic search, allowing users to explore a collection of TRs by combining search terms with ontology-based filters. Yet another analysis may look for drug-biomarker correlations in the TRs. BCE is analogous to the programming language level of representation. This level defines the program, and then various different analyses compile to different targets for different purposes, for example, running on different hardware. But one can always refer back to the program as written by the programmer, or in this case, the BCE as validated by the vetting process, as the agreed definition of what was expressed.

### Summary of constitutive hypotheses

Let us summarize the 10 constitutive hypotheses, in order to provide a point of reference going forward:

- H1. The Case Efficiency Hypothesis: A large number of coordinated case reports can be as useful in treatment validation as a smaller number of large scale trials, or even more so.

- H2. The Treatment Rationale Hypothesis: Treatment rationales, connecting observations to treatment decisions (pro or con), are the most important knowledge for decision support.

- H3. The Abundance of Outcomes Hypothesis: There are many sorts of direct and indirect outcome measures that can be useful in treatment validation, not just overall survival benefit.

- H4. The Expert Focus Hypothesis: It is efficient to start with current expert reasoning (as revealed in TRs), and branch out from there as necessary.

- H5. The Case Context Hypothesis: The case context, and, to the extent possible, surrounding discussion, are important in understanding TRs and their validity.

- H6. The Problem-Solving Tumor Board Hypothesis: Advanced Problem-Solving Tumor Boards are an efficient place to capture TRs.

- H7. The Coordination Over Collaboration Hypothesis: Coordinating individual treatment decisions across the whole oncology community is more efficient than physicians collaborating.

- H8. The Biomedical Controlled English Hypothesis: Biomedical Controlled English (BCE, a form of CNL) is an efficient and effective way to capture TRs.

- H9. The Nano-Publication Hypothesis: Vetted TRs should be published in an open access, archival, and API-accessible nano-publication-like setting.

- H10. The BCE Publication Hypothesis: In addition to being a good format for TR capture, BCE is the preferred form in which TRs should be stored and published.

The next section describes our prototype implementation of a Precision Oncology 3.0 platform, whose design is motivated by the above hypotheses.

### Prototype PO 3.0 platform overview

Implementing a rapid learning oncology system requires developing tools to capture and distill the case contextualized treatment reasoning related to each patient encounter, and rapidly translating these to support clinical decision making. This section describes our prototype implementation of a Precision Oncology 3.0 platform. Appendix provides a detailed walkthrough of the primary expected workflow through the prototype tools.

The prototype including the following components:

1. TrEx (the Treatment Explorer): An implementation of the analytical tool that ranks potential treatments, based on a putative patient’s biomarker profile, combined with TRs, as envisioned by Mocellin et al. and Stevovic et al.

2. Quark: A tool to capture case summaries and the important content of the discussion, focused on TRs, in BCE (CNL).

3. A vetting tool for rapid lightweight peer review of TRs.

4. An (unofficial) “Nano-Journal” where vetted TRs are published.

The remainder of this section explains these components, linking them, where relevant, to the hypotheses developed in the previous section.

#### Primary workflow

**Fig. 1:**
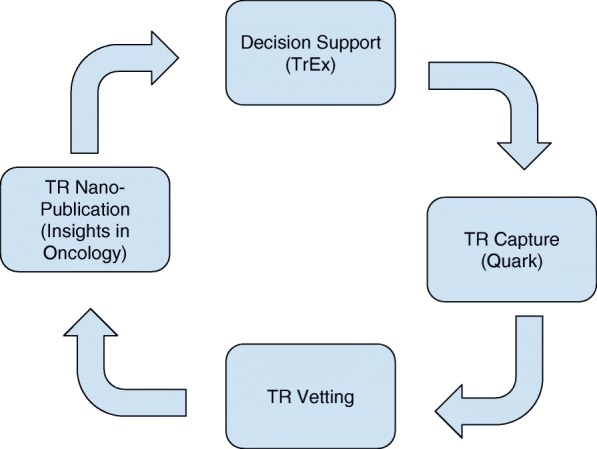
The envisioned rapid learning loop, supported by our prototype platform

Shrager and Tenenbaum [25] envisioned Precision Oncology 3.0 as an ongoing loop, depicted in Fig. 1.

Begin on the right of Fig. 1 with Quark, a tool for TR capture in a psTB context (H1-H6, and H8). Quark enables a trained “Clinical Analyst” (CA) to capture case summaries and associated TRs, represented in BCE, directly into a pre-vetting knowledge base. Note that these TRs are not entered into the master knowledge base yet because they are unvetted.

The vetting tool (bottom box in Fig. 1) supports various types of checking and scoring, ranging from checking de-identification and correct BCE encoding, through scoring the TR along various dimensions. In the present implementation we collect three-point scores along dimensions of agreement, generality, importance, and level of evidence. Based on informal interviews with professional oncologists, we learned that they themselves very rarely deal with specific quantitative information (except regarding dosing), but rather communicate among themselves in semi-quantitative terms, for example by describing the patient’s disease as “progressing”, “rapidly progressing”, “stable”, “in remission”, etc. Therefore, rather than trying to gather numerical measures along these dimensions, the vetting tools transacts entirely in three-point Likert-type scales. Specific user communities may find it useful to have different, finer scales.

Presumably different combinations of people and tools will undertake various aspects of vetting. We expect, for example, that de-identification checking would be mostly automatic, that BCE authorship would be carried out by trained domain experts who may well not be MDs and/or PhDs, but that scoring along content-relevant dimensions would usually be carried out by domain experts, likely MDs and/or PhDs.

Vetted TRs, represented in correct BCE, are also automatically compiled into an internal representation specific to the TTD application.

All of this information -- the BCE, the vetting scores, and the TTD-related internal representations -- are then published in the nano-journal (H9-H10; left hand box in Fig. 1). From there, these can be utilized in any number of ways. The particular way we have prototyped is in our TrEx tool (top of Fig. 1), which is a direct implementation of the Mocellin et al. algorithm. We envision that this tool would be used in decision-making contexts, such as tumor boards, closing the rapid learning loop, as envisioned by Shrager and Tenenbaum [25].

In accord with H5, the Case Context Hypothesis, all TRs maintain provenance linkages to their source. In the 2010 TTD, these were extracted from published papers, but the present TRs are collected from tumor boards. The prototype includes a means, everywhere that a TR appears, to inspect the provenance, such as the publication or tumor board transcript. Moreover, when citations are entered with TRs, we link to these through Google Scholar or pubmed (these are highlighted in the Appendix walk-through).

#### Details and issues

In the remainder of this section we briefly describe some details about the prototype implementation that are important, but are not theoretically motivated. This section can be skipped by those not interested in this level of detail. Appendix provides a complete walk-through of these prototype applications, and points out a number of additional features which are outside the scope of the present discussion. Readers may also explore the prototype themselves, using URls provide in the appendix.

##### Issues in Case and TR Capture

Quark is conceived as the biomedical analog of a court-reporter’s “steno-type” station. As described above, a specially-trained professional, that we call a “Clinical Analyst” (CA), would use Quark to capture case-contextualized TRs, from a setting such as a molecular tumor board (H6). TRs captured in this manner would be expressed by the CA in Biomedical Controlled English (BCE). In the best case, the clinical analyst is a medical scribe or fellow, or at any rate has enough understanding of the medical language to be able to translate from discussion to TRs in close to real time. As one can easily imagine, this may be extremely difficult, and one can envision any number of speech capture and read-back tools to aid in this process. However, as discussed in H8, we have chosen BCE as the final published representation, and so that is the primary modality supported in the present prototype. The difficulty of this task is made somewhat simpler by the knowledge that there is a vetting stage where experts can check de-identification, BCE encoding, and assess the validity of TRs. Some of these checks may eventually be automated, or at least tools built to help the CA in this task.

Initially, Clinical Analysts will have to be trained to record in BCE. Although pilots live in an environment where they are explicitly taught to speak the CNL that is “pilot-speak” [20], the same is not the case for physicians. Of course, all communities create semi-formal languages within their domain, and this is the case for medicine as well. (Watchers of the TV program ER, for example, will recognize many examples of common phrases that doctors are yelling at one another, such as: “trauma panel”, shorthand for a series of standardized tests.) But there is, as yet, as far as we are aware, no such widespread semi-formal language for communicating Treatment Rationales. As we are proposing that TRs be coded in BCE for which where there is, at the moment, no natural standardized controlled language, we expect there to be a period where trained analysts will have to translate from tumor board discussion into BCE. However we hope that after many examples of such translation have happened, and if physicians find tools such as ours useful, the intelligence of the tools, and the training of the speakers will converge to the point where it becomes as natural as pilot-speak is for pilots and controllers.

##### Issues in Vetting

Aviation communication practices have evolved procedures to validate that the communications is correctly transmitted and understood. For example:

“Acknowledge all ATC (RADAR) instructions with a readback. [...] Readback all frequencies, X-ponder codes, and headings. Include the the direction of the turn to the heading just to be certain. Occasionally the turn is required to take the long-way-around for spacing. Query ATC if in doubt” [20].

Similarly, we have created a vetted process that takes place before TRs are shared among participating psTBs. Vetting is intended to be lighter and faster than peer review, and allows for rapid information exchange of TRs in a controlled setting, for example among participating institutions. Stricter formal peer review would likely be applied before public release of any knowledge from this network.

We envision that there will be both local and central standards for case+TR vetting and release. There are many additional questions that we have not addressed as to, for example, whether or not the vetting is anonymous, how many agreements one needs before release, and so on. In the prototype implementation we have made a “best guess” about the level of vetting that might be typical, and about the scoring dimensions. However, these will almost certainly end up being chosen differently by the administrators of a given deployment.

##### TrEx Statistical Issues

The TTD algorithm described by Mocellin et al. [16] already takes into account the type of model (e.g., in vitro, in vivo, in human, clinical) as well as the number of cases (e.g., 1 for a case report, or the number of total cases for a clinical trial or meta-analysis). Each TTD entry coming from a case report (e.g., from a tumor board via Quark) represents a new row -- a TR in our present terminology -- and will have Model = human/clinical, but only one case (Cases = 1). Put another way, a case report is, to TrEx (which implements the TTD algorithm) just a clinical series with a single patient (*n* = 1). Although by virtue of the small n (=1), each such case report contributes very little to the overall statistics, the Case Efficiency Hypothesis (H1) holds that many such *coordinated* reports will have a cumulative effect. A statistical independence problem arises if we include multiple TRs from the same person (or paper), so in the present work we assume that all TRs are independent. In practice this assumption will not hold, and needs to be addressed.

##### Issues in BCE compilation

The range of BCE handled by our prototype implementation is quite narrow, focused almost uniquely on what is needed for TrEx. As a sort of “hard line” experiment, we chose in the present implementation to use a very simple open source language parser; Indeed, the parser we used is almost line-for-line out of Peter Norvig’s Principles of AI Programming [18], called “simple2” [19]. The core of the parser is one Lisp function, called extend-parse, that is only 17 lines long. (Lisp code actually doesn’t have code lines as such; 17 is the count of code lines covered by that function in that particular file.)

Of course the grammar for the TR application is much larger than the parser code, yet it is not outrageously complex, comprising a few dozen rules, and the ontology comes from the NCI Thesaurus, so even just a few dozen rules can correctly understand a wide range of BCE TRs, as complex as:

- EIF2AK3-alk fusion in NSCLC confers sensitivity to ceritinib, as demonstrated in a randomized controlled trial with 200 patients with stage 4 lung cancer (Won, Mambetsariev, and Salgia, BMC Cancer. 2016 Aug 2;16:568)
- This 50 year old male’s stage 4 NSCLC tumor was apparently sensitized to ceritinib by an EIF2AK3-alk fusion.

As well as a wide range of simpler forms.

The reason that we chose to manually create a grammar for this prototype, as opposed to applying any number of available machine learning methods, is simply that, at the moment, we do not have enough examples to train a grammar to the level that we can write one by hand. We expect that, in the fullness of time, this balance would shift to having enough examples to train a grammar that performs as well as our hand-crafted one.

##### Ontological Issues

The ontology utilized in this prototype is the NCI Thesaurus. The platform can “understand”, i.e., has a unifying code for, a wide range of diagnoses and treatments, as well as their synonyms. However, because the BCE is conceptually open-ended, and the intended use case is advanced cancers at top psTBs, it is quite likely that TRs will arise that include non-standard vocabulary (or at least vocabulary that does not appear in the NCI Thesaurus). An excellent example of this comes from alternative therapies, especially those based on changes to a patient’s diet. Although alternative therapies that are unique, for example, “acupuncture”, do appear in the NCI Thesaurus, as do many foods, such as “blueberries”, many common treatments with peer-reviewed clinical support, such as “plant based diet” [6], do not appear in the thesaurus. This problem extends as well to observables. Whereas most common genomic aberrations are in the NCI Thesaurus, and unusual ones can be understood by a relatively simple grammar, “non-technical” characteristics, such as patient preferences, type of insurance coverage, and many other important features, are not in the NCI Thesaurus. To handle these sorts of language, our grammar is open-ended, and tries to assign a rough part-of-speech to every word and phrase in the input, even if no specific ontological binding can be found. Unfortunately, this is very difficult for the simple bottom-up extension parser that we are using; It is better handled by a top-down, or trained, predictive parser.

##### Universal API-Readiness

Although there are user interfaces (UIs) provided to explore the prototype platform tools, anyone who goes through the walk-through in Appendix will observe the rough state of these UIs. We envision that the various services underlying these particular UIs will actually be used by many applications aside from those that we happen to have built, and that they will have their own UIs. Therefore, every service in the prototype platform was built first with an Applications Program Interface (API), and then the UIs were added primarily for demonstration purposes. These APIs are documented in the platform documentation.

## Discussion

Conventional, or even modern adaptive clinical trials, are no match for the immense dimensionality of the cancer problem. Rapid Learning Precision Oncology, or Precision Oncology 3.0, is predicated on the hypothesis that it is more efficient to operate a system of coordinated individual treatment experiments across the entire cancer community, than it is to laboriously mount clinical trials. To carry out this vision requires that the community of physicians, and other oncology researchers and professionals, be able to efficiently learn from the treatment reasoning that takes place in the thousands of clinical encounters that occur daily across the community, but which are rarely reported in the literature. Moreover, these experiences must be coordinated in order to rapidly validate successes and rapidly quash failures.

We have focused here on the capture, and rapid vetting and dissemination of Treatment Rationales (TRs), a case-contextualized subset of expert reasoning. Coordination aside, even just capture is a challenge, as there is today no rapid and highly accessible case reporting system. Even at major cancer centers, where difficult cases are often considered by molecular tumor boards, these boards usually do not have any organized way of recording their own cases. As a result, the most up-to-date knowledge travels by word-of-mouth, or the writing of case reports and slow, journal-based communications. Additionally, some of the most important knowledge is often lost completely, usually not making it even into the patient’s medical record. Although this knowledge often appears in published case reports, a vanishingly small number of cases are reported in print. In the best case the TRs would be captured from difficult clinical cases discussed in the psTBs at top cancer centers. The capture, coding for both human and machine interpretation, and rapid dissemination of TRs should benefit numerous projects focused on advanced cancer. Even aside from the particulars of our prototype applications, such as TrEx, the ability to search, query, and aggregate case-contextualized TRs could enable other important applications, such as, case series aggregation, reimbursement support based upon treatment rationales, and expert identification and referral.

### On big data

Most biological data capture projects are focused on either data that is in the EHR already, or augmenting this data with additional “Big Data” such as whole-genome or expression sequencing. Our approach is complementary to these. We believe that focusing only on the “big data” aspect of medicine is inefficient because the dimensionality of the problem is very high, and the cost of data gathering (either in economic or ethical terms, e.g., pain and suffering, perhaps even death) is extremely high. As a result, the number of independent observations (sometimes referred to as the sample size, or ‘n’ of a study) is generally much smaller than its dimensionality, that is, the number of features. Put more simply, there’s lots of data about very few patients. Whereas the typical “big data” problem has many samples (a large number of independent observations, large ‘n’) for relatively few features (dimensions) -- biomedical “big data” generally has many features (high dimensionality) for very few samples (small n). Hypothesis formation this sort of data setting requires a different approach than for the typical big data setting. There are many ways that data can be “folded” to reduce its dimensionality. For example, the names representing genes, enormously reduces the 3 billion bases of DNA sequence information to around 20,000, and this can be reduced even further by noting only aberrations, using pathway models to relate features to one another, or empirically discovering correlations between features. Our expert-sourced, case-contextualized TR approach gathers a novel category of knowledge that can feed into the folding of the features space, and thereby efficiently reduces the dimensionality of the problem, while at the same time increasing the sample size.

### On the literature

Although is it inefficient, the peer-reviewed literature will, for the foreseeable future, serve as the touchstone of vetted results. We have not focused on this path here because many other projects, such as IBM’s Watson, are working on knowledge extraction from publications. Similar to our stance with respect to (typical) Big Data, nothing in our implementation precludes this path, and, indeed, the TTD [16] relies exclusively on it, although manual extraction is not scalable (and we have our doubts that AI is up to this sort of highly summarized extraction, in part based upon the “dynamic” knowledge hypothesis, discussed above in H5). One idea that could be scalable and aligns with the “expert-sourcing” model that we rely upon (H4) is to ask the authors of the papers themselves to summarize the Treatment Rationales in their papers, as was done by Rappaport et al. [21]. Indeed, we hypothesize that the efficiency of literature-based knowledge extraction will be greatly improved by only bothering to process papers that are explicitly mentioned in psTBs.

### On coordination

Capture and rapid dissemination of case-contextualized TRs is not sufficient to efficiently search the huge space of cancer treatments crossed with disease characteristics. The Case Efficiency Hypothesis (H1), and the GCTA process proposed by Shrager and Tenenbaum [25] emphasize that treatment decisions must also be *prospectively, adaptively coordinated* across all patient encounters. Prospective adaptive coordination (a) avoids unnecessary replication of either positive or negative experiments, (b) maximizes the amount of information obtained from every encounter, and (c) permits efficiently testing causal hypotheses. Fiore et al. [5] demonstrated that this sort of prospective, adaptive coordination can be efficiently carried out across the Veteran’s Administration because they are connected by a common EHR system. Carrying out this sort of coordination is the intended end game of the current platform. However even in its prototype form, figuring out how to carry out prospective, adaptive coordination in the present TR-based system, with many more potential treatments and biomarkers is far more complex than the sort of three-arm trial described by Fiore et al. [5]. Our next phase of development will be to explore ways to infuse this sort of prospective, adaptive coordination into the prototype. One idea of how this might be done is to re-sort the tests in TrEx to reflect the GCTA prospective information value of each test, but there are many other ways that prospective, adaptive coordination could be integrated into the current prototype.

### Related work on Knowledge Capture from Tumor Boards

How medical professionals think has been of interest for at least 40 years (e.g., [4]), and tumor boards are commonly recognized as an important locus of such problem solving [3]. The prototype platform described in this paper provides a means of capturing, vetting, and creating a global database of expert-sourced, case-contextualized Treatment Rationales from settings such as tumor boards, and of using this knowledge in decision support. In accord with Hypothesis 6, our work has been focused on “problem-solving Tumor Boards” (psTBs) as a convenient setting in which clinical problem solving is exposed, and can be conveniently captured. To our knowledge, the importance of tumor board discussion as a direct source of data and knowledge has not been previously recognized.

There are a growing number of products aimed directly at tumor boards, however, most of these are focused on data, not knowledge. Syapse, for example, offers a product focused on cancer data capture, management, and analysis, with the goal to “...streamline the Molecular Tumor Board Collaboratively determine and disseminate treatment recommendations based on the full scope of patient data available.” [27]. Consistent with this focus on data, Syapse does not deal at all (so far as we can tell) with treatment reasoning.

Roche’s Navify tumor board product [17] is an exception to the pure data focus of products such as Syapse. Like our work, Navify explicitly supports the capture of treatment reasoning. However the goals of capturing treatment reasoning within Navify are somewhat different from ours. Whereas our goal is to explicitly support future treatment decision making, Navify’s goal is “*ensuring the decisions get fully implemented at the point of care*. *The patient’s oncology care team can refer to these decisions and the rationale behind them later to ensure they are following the prescribed plan.*” [emphasis ours]. This suggests that the Navify use cases focuses on the treatments recommended by the board and their rationale. To the contrary, in accord with hypothesis 2, we are concerned with a larger set of decisions and reasoning, especially including “contra-recommendations”, treatment hypotheses that are *rejected (or ranked lower)*, either because they are incorrect or infeasible. We believe that the reasoning behind contra-recommendations can provide important knowledge about potential novel hypothesis, and/or about why certain treatments are incorrect or infeasible. This sort of knowledge would *never* show up if one was only focused on the recommended treatments. Again, to our knowledge the importance of contra-recommendation reasoning has not been previously recognized.

## Conclusion

The prototype platform described in this paper provides a means of capturing, vetting, and creating a global database of expert-sourced, case-contextualized, Treatment Rationales from settings such as problem-solving (e.g., molecular) tumor boards, and of using this knowledge in decision support. This work embodies the closed oncologic rapid learning process that Shrager and Tenenbaum [25] called “Precision Oncology 3.0”. The exercise of development forced us to be explicit about many hypotheses and assumptions that were previously underdeveloped in prior proposals. These are likely to be helpful in informing design and implementation decisions in similar projects, and form the basis of our ongoing work in GCTA treatment coordination.

We have no illusion that this sort of complex multi-application platform will fit smoothly into the existing oncological workflow. As Berg [1] among other have made clear, even simple computational applications often face a significant energy barrier. The analogy to Air Traffic Control [23] brings this into stark relief: Commercial Aviation is interestingly “quasi-military” in the sense that everyone follows the procedures, which were, for the most part, developed by the aviation community itself in recognition of the importance of safety; If pilots don’t collaborate well with controllers, people may die, indeed the pilots themselves may die! People die in oncology also, but, unlike pilots, oncologists do not have an Air Traffic Control system to help monitor and organize the chaos, and so physicians have not grown up in the sort of quasi-military culture that pilots have. We believe that the near term will see this beginning to change -- that medical professions, as well as the commercial organization that operate in concert with them, such as pharmaceutical companies, device manufacturers, hospital systems, and even payers, will begin to operate in greater concert through technologies such as the platform we have described. There are already existence proofs of the possibility of this future. The Veterans Administration, for example, has operated for nearly three decades with the VistA computer platform [29], and this has been used recently to operate advanced, adaptive “Point of Care” trials [5]. Yet Vista does not implement the tight, rapid-learning knowledge-loop envisioned by Shrager and Tenenbaum [25], Shrager [22, 23], where, like pilots and controllers, physicians interact to efficiently share knowledge through controlled language capture that goes through immediate vetting and feeds back to the ongoing workflow through decision tools. We think that that will require more fundamental cultural, technical, and procedural changes, and hope that the present work can serve as both a conceptual and technical prototype for that possible future.

## Availability and requirements

**Project name**: ccPO30ProtoypePaperRelease.

**Project home page**: https://github.com/jeffshrager/ccPO30ProtoypePaperRelease

**Operating system(s)**: Platform independent (requires Lisp and Javascript).

**Programming language**: Lisp and Javascript.

**Other requirements**: Various lisp packages available via QuickLisp.

**License**: MIT Open Source.

**Any restrictions to use by non-academics**: n/a.

## Figures and Tables

**Fig. 2:**
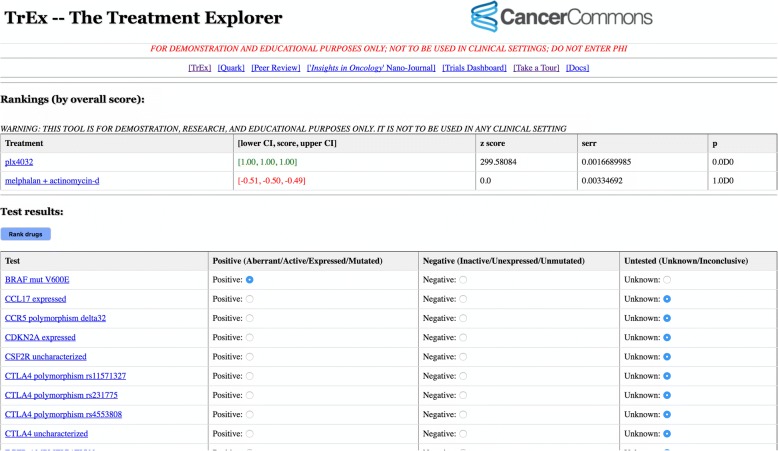
TrEx -- The Treatment Explorer. The user selects from among possible observations in the lower table, and the Mocellin algorithm ranks treatment hypotheses in the top table

**Fig. 3:**
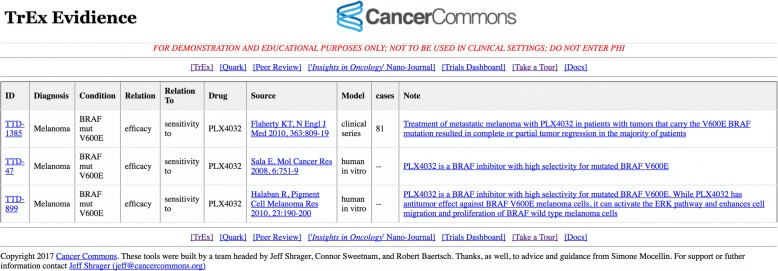
TRs that participate in ranking PLX-4032

**Fig. 4:**
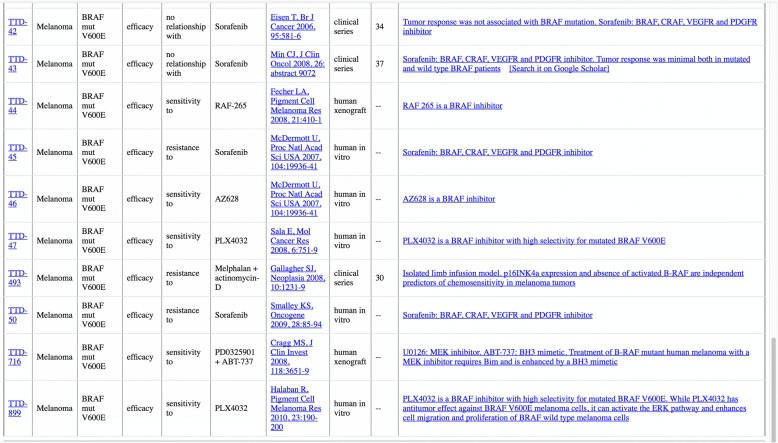
TRs related to BRAF V600E mutation

**Fig. 5:**
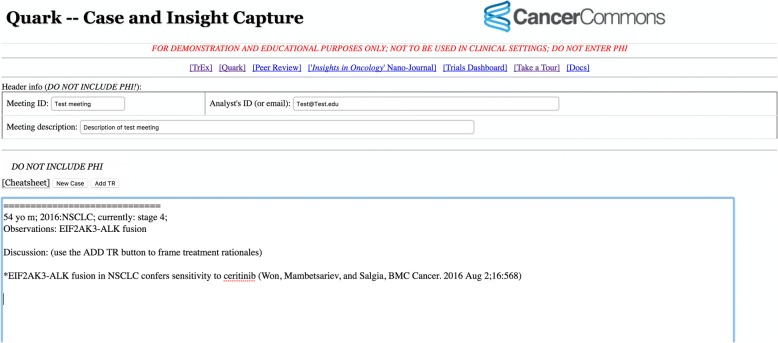
The Quark Analyst’s Dashboard

**Fig. 6:**
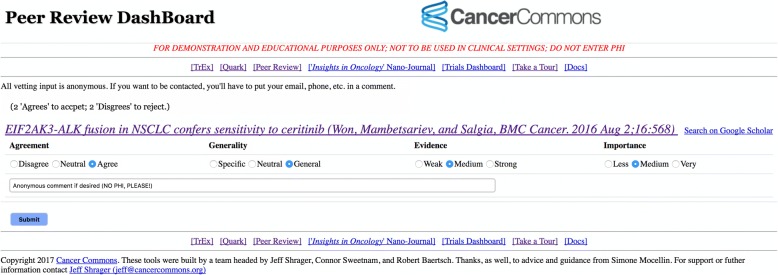
the TR Vetting tool

**Fig. 7:**
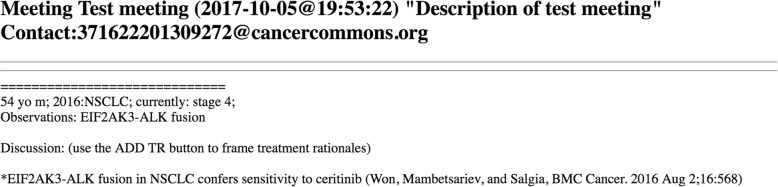
Case context for the EIF2AK3-ALK fusion TR

**Fig. 8:**
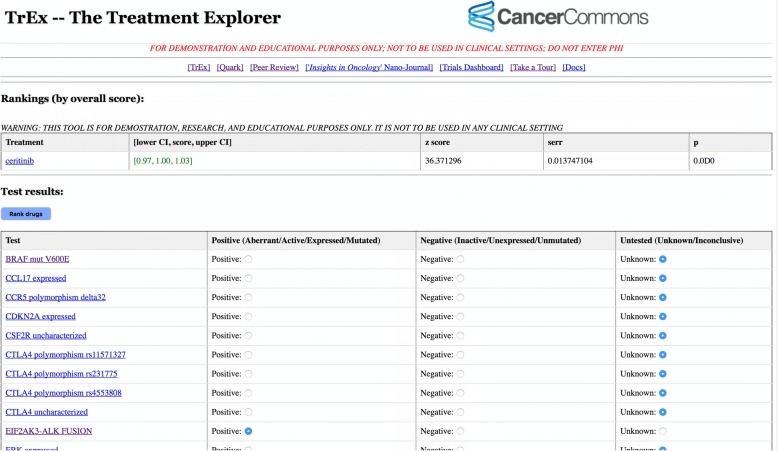
TrEx with the vetted EIF2AK3-ALK fusion TR

**Fig. 9:**
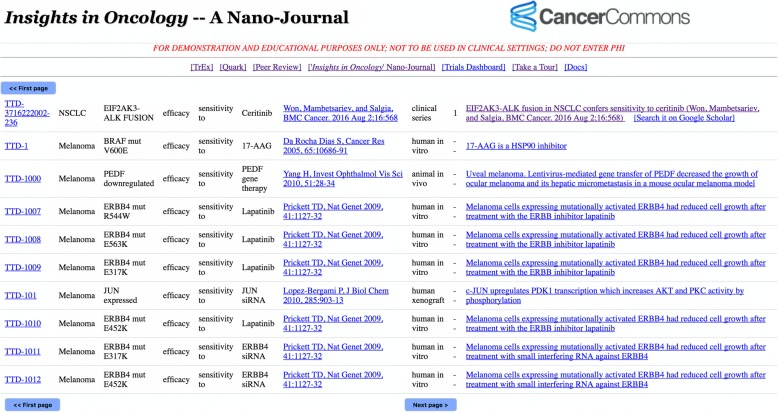
The (prototype) “Insights in Oncology” Nano-Journal

## Appendix

### A Tour of the Precision Oncology 3.0 Platform

The platform examined in this tour is intended to support an ongoing rapid learning loop, as discussed in the main paper (Fig. 1). However, having to start somewhere in the loop, we begin with the decision support tool, TrEx, The Treatment Explorer. Because the sort of person envisioned as the user of these tools varies, we will explicitly change hats a number of times.

### TrEx -- The Treatment Explorer

TrEx is an implementation of the TTD algorithm of Mocellin et al. [16]. The principal function of TrEx is to rank possible therapies based upon test results, combined with peer-reviewed evidence. The TrEx prototype here uses the database published in that paper, with some minor simplifications. Therefore, the particular treatments that you will see in this tour are out of date.

We begin by putting on our oncologist (physician) hat, and opening the TrEx prototype application at the below URL, or or see the code at the below GitHub repository:

http://vtb.xcures.com:4240/trexui

https://github.com/jeffshrager/ccPO30ProtoypePaperRelease

You will see a table of tests, with “Positive”, “Negative”, and “Untested” buttons to the right of each. Focus on the first test in the list: “BRAF mut V600E”. To begin with, click “Positive”, to the immediate right of that test, and then click the button just above the table, labeled “Rank Drugs”. A new table appears above the test table, containing two treatment options (Fig. 2): PLX4032 (now called Vemurafenib), and the combination treatment melphalan + actinomycin-d. Find the BRAF V600E test again in the tests table. It will be marked “positive” (as you had left it). Change the setting to “negative”. Again rank the treatment options, and notice that, as expected, the order of the two treatments is reversed.

Explore TrEx a bit by setting some of the test results in various ways. Notice how the treatment rankings change. The ranking algorithm is described in detail in the Mocellin et al. [16] paper.

#### Drilling Down to the Treatment Rationales

Notice that all of the tests and treatments are clickable links. Return to the PLX4032 treatment, in the upper table, and click that link. You should see three rows of evidence appear in a new browser tab (Fig. 2). These are the three sources of evidence that participate in the ranking of this treatment. We call each TrEx record a “Treatment Rationale” (TR). This is essentially the same as what Mocellin et al. [16] called a “Treatment Hypothesis”. The meaning of these fields is explained in that paper.

The user can “drill down” either from a condition (i.e., aberration, as Fig. 3) or treatment hypothesis to the TRs (Fig. 4), and drilling further, by clicking on the “source” or “notes” fields will open the appropriate web page at PubMed.

Return to the main TrEx page and find the BRAF V600E mutation test line. Click on the “BRAF mut V600E” link. This should again bring up an evidence table (in a new tab), this time with many rows (Fig. 4). These are the rows of evidence related to this clinical observation (that is, the observation of a BRAF V600E mutation).

Notice that many possible drugs besides PLX4032 appear in this table, but only two of them are ranked when this aberration is observed. This happens because only the PLX4032 and the melphalan + actinomycin-d cocktail entries have (a) clinical evidence (i.e., most of the other evidence is in vitro, which is not counted as strong enough to be included in this implementation of TrEx), or (b) the relationship was experimentally null (i.e., it reads: “no relationship with” in the “relation to” column). To see this, look for “Sorafenib” in the “Drug” column of Figure 4. There are a number of rows providing evidence regarding sorafenib, yet it does not appear in the rankings when a V600E transition is observed (selected as positive). This is because every row that relates to Sorafenib is either non-clinical-level data (in the “model” column), or, where there is clinical evidence, it says “no relationship with” in the “relation to” column. TrEx is a direct implementation of Mocellin et al.’s algorithm, with almost no changes. The only thing changed is that the only evidence allowed to participate in treatment ranking is that arising from clinical models (case series, controlled trials, or meta-analyses of controlled trials).

#### Drilling Down to the Evidence

Notice that the “source” and “notes” column entries in this evidence table are also clickable. Clicking these will (in most cases) bring the user to the PubMed entry for the paper from which the evidence was obtained. This enables one to examine the source of the evidence if one likes. We shall see that when the evidence is a case context, these links display the case summary.

#### Knowledge Base Cleanup

Before leaving TrEx, scroll all the way to the bottom, and click the button labeled “Reset DB”. This will remove left-over TRs from your, or others’, previous experiments. (This will also delete the data created by anyone else who happens to be using these prototype application at the same time as you are. Hopefully this will not be a common coincidence. In real usage, users would be logged into the system with permission controls, and functions such as this would only be available to system administrators.) Scroll up to the place where “EIF2AK3-ALK fusion” tests would appear if there were any (the table is alphabetical), and notice that there are no EIF2AK3-ALK fusion tests. (The TTD used here was created for melanoma, and in 2010 there was no clinical evidence relating EIF2AK3-ALK fusions to melanoma.)

#### Quark: A Tool to Capture Case-Contextualized TRs in Biomedical Controlled English

Mocellin et al. [16] manually created the table of TRs for melanoma, which they called the “Targeted Therapy Database” (TTD). We have argued, after Stevovic et al. [26] and Shrager and Tenenbaum [25], that the knowledge base should be continually updated by adding case-contextualized TRs captured from problem-solving Tumor Boards (psTBs). This is the role of the Quark tool.

Quark was conceived as the biomedical analog of a court-reporter's steno-type machine. A specially-trained professional, that we call a “Clinical Analyst” (CA), would use Quark to capture case summaries and Treatment Rationales in a setting such as a problem-solving tumor board. TRs captured in this manner are expressed by the CA in Biomedical Controlled English (BCE), a version of a Controlled Natural Language (CNL). The TRs, thus captured in BCE/CNL are then vetted, i.e., subjected to lightweight peer review. Those TRs that pass vetting are immediately added to the TrEx database, and will have immediate impact on the drug ranking results as computed by TrEx.

To get from TrEx to Quark, use the navigation bar near either the top or bottom of the page. And while it's changing pages, quickly switch into your Clinical Analyst's hat!

Figure 5 depicts the Quark Analyst's Dashboard, prefilled with some example notes from an imaginary tumor board meeting. We suggest entering something recognizable into at least one of the fields at the top of the page. This will enable you to identify that the transcript is yours if you were to see it again. (Do not enter personally identifiable information, such as your name!)

Now let's examine the imaginary tumor board transcript. First, notice the header labeled “[Cheatsheet]”. If you hover over this link (at either the top or bottom of the transcript) a large “tool tip” will appear, reminding you -- in the role of Clinical Analyst for this tumor board -- how Quark expects to see certain special entries. The only one we care about for the moment is where “insights” are prefixed by asterisks (*). (For the present discussion, we consider insights and TRs to be the same thing.) The lines in the transcript prefixed with asterisks are TRs represented in the Biomedical Controlled English (BCE), as described in the paper.

Let's preview what's going to happen next, before actually doing it: We will push some of the TRs in this imaginary tumor board transcript into TrEx, and see that they change the way that TrEx ranks treatments. The TRs that we're going pushing through in this way are written in BCE. When we tell Quark that we're done with this transcript, the system will translate the TRs (indicated by asterisks in the first column) into the internal representation used by TrEx. It then submits these to vetting (i.e., lightweight peer review). In order for the TRs to actually get into the knowledge base, two reviewers have to agree with them. (This requirement is settable to the taste of particular communities.) Vetted TRs are added to the TrEx knowledge base, where they participate in the TrEx drug ranking process.

Begin this process by scrolling to the bottom of the Quark page and clicking “Save and Close”. You will be prompted to confirm this by clicking “ok” or “yes” when the modal dialog pops up.

#### A Tool for Rapid Vetting of Case-Contextualized Treatment Rationales

When you Save and Close the Quark page, you will be put into the TR Vetting tool (Fig. 6). As mentioned above, you now need to be wearing both your Clinical Analyst and your Oncologist (physician) hats.

Find the entry containing the TR above:

- EIF2AK3-alk fusion in NSCLC confers sensitivity to ceritinib (Won, Mambetsariev, and Salgia, BMC Cancer. 2016 Aug 2;16:568)

Our job as a vetter is to assess this assertion along the dimensions indicated by the four 3-way Likert-type scales below the assertion: Agreement, Generality, Evidence, and Importance. Focus on the leftmost scale, labeled “Agreement”.

Suppose that we actually don't know whether we agree with this assertion or not because we don't know enough about it, or the context in which it was collected. There are links in the vetting tool that can help us understand the assertion better.

Start by clicking on the assertion itself. This will bring up a tab containing a read-only display of the Quark transcript that we had just submitted (Fig. 7).

If you filled in some of the fields, you should be able to tell that this is the one you actually saved. This can be useful in understanding the context in which this assertion was made. Note that the email address is anonymized, so that regardless of what was entered, all that any viewer sees is a long number @CancerCommons.org. This permits a reader to contact the author of the case (the CA) by emailing to that address, which will be forwarded by the platform to the CA who authored the case transcript. The recipient can decide whether to open an non-anonymous conversation with the person asking the question.

Now go back to the vetting page, and notice that there is a button labeled “Search on Google Scholar”. When you click this, the whole text of the TR is entered into google scholar and searched.

These tools should help you, in the role of a vetter, figure out whether you believe the assertion or not. Let's say that we decide that we do believe the assertion. Select “Agree”, and then scroll to the bottom of the page and click “Submit”. This will take you back to TrEx.

Unfortunately, it's a bit premature to celebrate, because you will recall that we need two reviewers to agree with the assertion in order for it to be accepted and added to the knowledge base. For this tour we are, of course, going to fake it by clicking the link on the nav bar called [Peer Review]. This will bring you back to the vetting page. Click “agree” again on our favorite TR, and then “Submit” again. This time we'll be in TrEx with that TR (hopefully) added to the knowledge base! (If you were to once again return to the vetting page, you would find that the TR of interest no longer appears in the vetting interface, because it has already been accepted, and requires no more vetting.)

### Closing the Loop: Back to TrEx with Quark-captured Treatment Rationales

We should now be in a new world where TrEx knows about EIF2AK3-alk fusion and its relationship to ceritinib. Recall that above, when we reset the database, there was no entry among the tests for an EIF2AK3-alk fusion test. Having posted, and vetted, a TR relating EIF2AK3-alk fusion to ceritinib, this should now appear among the tests. Check that out by finding the row in the appropriate row in the test table (Fig. 8). Clicking on this new test entry will bring up the appropriate evidence table, and you will observe that this came from a vetted TR that was translated from the BCE appearing in our imaginary tumor board transcript.

Recall that when the evidence came from papers, you could click through to the publication entry in PubMed. When the evidence comes through Quark, these functions work similarly to the Vetting tool: You can click through to either the Quark transcript context, or to a Google Scholar Search.

#### The Insights in Oncology Nano-Journal

As described in the body of the paper, all the insights in the knowledge base, including those that you just added (and those that will eventually be added by real CAs) are published in an archival “nano-journal” that we call “Insights in Oncology”. (This is a working title; We're pretty sure that this is already taken, and we will eventually need a new name.)

Navigate to [‘*Insights in Oncology*’ Nano-Journal] from the navigation bar (Fig. 9).

At the moment the nano-journal is just a reverse chronological list of all the treatment rationales in the knowledge base. There isn't much you can do here other than scan forwards and backwards in this list, and click through to the papers on PubMed or the Quark case contexts, just as we did before when we were looking at TrEx records from the TrEx UI. Eventually we will provide functionality such as search and commenting.

## Abbreviations

BCE: Biomedical Controlled English
CA: Clinical Analyst
CC: Cancer Commons
CNL: Controlled Natural Language
EMR: Electronic Medical Records (System)
psTB: problem-solving Tumor Board
TR: Treatment Rationale (or Reasoning)
TrEx: Treatment Explorer

## Footnotes

1 There are many kinds of tumor boards. Ranging from quality control TBs, which ask whether guidelines are being followed, up through what we are called psTBs. psTBs are sometimes called “Molecular Tumor Boards” (MTBs), but this is a misnomer as they consider any useful information, often going well beyond the molecular details of the case.

## References

[1] Berg M (1997). Rationalizing medical work decision-support techniques and medical practices.

[2] Controlled_language: https://simple.wikipedia.org/wiki/Controlled_language. Accessed 13 Oct 2017.

[3] El Saghir NS, Keating NL, Carlson RW, Khoury KE, Fallowfield L (2014). Tumor Boards: Optimizing the Structure and Improving Efficiency of Multidisciplinary Management of Patients with Cancer Worldwide. ASCO ASCO EDUCATIONAL BOOK.

[4] Elstein AS, Shulman LS, Sprafka SA (1978). Medical problem solving: an analysis of clinical reasoning.

[5] Fiore LD, Brophy M, Ferguson RE, D'Avolio L, Hermos JA, Lew RA, Doros G, Conrad CH, O'Neil JA, Sabin TP, Kaufman J, Swartz SL, Lawler E, Liang MH, Gaziano JM, Lavori PW (2011). A point-of-care clinical trial comparing insulin administered using a sliding scale versus a weight-based regimen. Clin Trials.

[6] Frattaroli J, Weidner G, Dnistrian AM, Kemp C, Daubenmier JJ, Marlin RO, Crutchfield L, Yglecias L, Carroll PR, Ornish D (2008). Clinical events in prostate cancer lifestyle trial: results from two years of follow-up. Urology.

[7] ICAO A38-8. Proficiency in the English language used for radiotelephony communications: International Civil Aviation Organization. https://www.icao.int/safety/lpr/Documents/A38.8.pdf. Accessed 13 Oct 2017

[8] Ioannidis JPA (2005). Why most published research findings are false. PLoS Med.

[9] Jaeger ME, Rosnow RL (1988). Contextualism and its implications for psychological inquiry. Br J Psychol.

[10] Knorr-Cetina KD (1981). The manufacture of knowledge.

[11] Kuhn T (2014). A survey and classification of controlled natural languages. Comput Linguist.

[12] Kuhn T, Barbano EP, Nagy ML, Krauthammer M (2013). Broadening the scope of Nanopublications.

[13] Kuhn T, Chichester C, Krauthammer M, Queralt-Rosinach N, Verborgh R, Giannakopoulos G, Ngonga Ngomo A-C, Viglianti R, Dumontier M. Decentralized provenance-aware publishing with nanopublications. Peer J Comp Sci; 2016. 10.7287/peerj.preprints.1760v1.

[14] Lyden PD, Meyer BC, Hemmen TM, Rapp KS (2010). An ethical hierarchy for decision making during medical emergencies. Ann Neurol.

[15] Mitchell TM, Keller RM, Kedar-Cabelli ST (1986). Explanation-based generalization: a unifying view. Mach Learn.

[16] Mocellin S, Shrager J, Scolyer R, Pasquali S, Verdi D, Marincola FM (2010). Targeted therapy database (TTD): a model to match Patient's molecular profile with current knowledge on Cancer biology. PLoS One.

[17] Navify: https://www.navify.com/tumorboard/. Accessed 13 Apr 2018.

[18] Norvig P. Paradigms of artificial intelligence programming: case studies in common lisp. San Mateo: Morgan Kaufmann; 1991.

[19] Norvig, P. http://norvig.com/paip/syntax2.lisp. Accessed 20 Oct 2017.

[20] Pilot Friend: IRF Radio. http://www.pilotfriend.com/training/flight_training/nav/ifr_radio.htm. Accessed 22 Sept 2017.

[21] Rappaport AT, Adamson DR, Shih L, Smith RG, Tenenbaum JM, Khoo B, Cho S, Wolff AC, Carlson RW, Whippen DA (2004). Smart search and analysis of ASCO abstracts: the 2003 ASCO pilot breast Cancer information exchange (BCIE) project. J Clin Oncol.

[22] Shrager J. Theoretical Issues for Global Cumulative Treatment Analysis (GCTA). Cornell University Library, 2013, http://arxiv.org/abs/1308.1066.

[23] Shrager J (2016). Precision medicine: fantasy meets reality. Science.

[24] Shrager J, GCTA: Global Cumulative Treatment Analysis. https://www.youtube.com/watch?v=p0ua9sMK6V4. Accessed 20 Oct 2017.

[25] Shrager J, Tenenbaum JM (2014). Rapid Learning Precision Oncology. Nat Rev Clin Oncol.

[26] Stevovic J, Maxhuni A, Khaghani-Far I, Shrager J, Convertino G, Gobbel R. Adding Individual Patient Case Data to The melanoma targeted therapy advisor. Presented at the 7th international conference on pervasive computing Technologies for Healthcare. Venice; 2013. https://ieeexplore.ieee.org/stamp/stamp.jsp?tp=&arnumber=6563891.

[27] Syapse Network: https://www.syapse.com/product/syapse-network. Accessed 13 Apr 2018.

[28] Velterop J (2010). Nanopublications: The future of coping with information overload. LOGOS J. world B. Community.

[29] VistA History: Overview. http://worldvista.sourceforge.net/vista/history/. Accessed 25 Oct 2017.

[30] Youngs N, Penfold-Brown D, Bonneau R, Shasha D (2014). Negative example selection for protein function prediction: the NoGO database. PLoS Comput Biol.

